# Preparation of Porous Carbon Nanofibers with Tailored Porosity for Electrochemical Capacitor Electrodes

**DOI:** 10.3390/ma13030729

**Published:** 2020-02-05

**Authors:** Jisu Kim, Youn-Ji Heo, Jin-Yong Hong, Sung-Kon Kim

**Affiliations:** 1 School of Semiconductor and Chemical Engineering, Jeonbuk National University, 567 Baekje-daero, Deokjin-gu, Jeonju-si, Jeollabuk-do 54896, Korea; okjisu@naver.com; 2 Carbon Industry Frontier Research Center, Korea Research Institute of Chemical Technology (KRICT), Daejeon 34114, Korea; yjheo@krict.re.kr; 3 School of Chemical Engineering, Jeonbuk National University, 567 Baekje-daero, Deokjin-gu, Jeonju-si, Jeollabuk-do 54896, Korea

**Keywords:** electrochemical capacitor, energy storage, carbon nanofiber, porosity, polyacrylonitrile

## Abstract

Porous carbon electrodes that accumulate charges at the electrode/electrolyte interface have been extensively investigated for use as electrochemical capacitor (EC) electrodes because of their great attributes for driving high-performance energy storage. Here, we report porous carbon nanofibers (p-CNFs) for EC electrodes made by the formation of a composite of monodisperse silica nanoparticles and polyacrylonitrile (PAN), oxidation/carbonization of the composite, and then silica etching. The pore features are controlled by changing the weight ratio of PAN to silica nanoparticles. The electrochemical performances of p-CNF as an electrode are estimated by measuring cyclic voltammetry and galvanostatic charge/discharge. Particularly, the p-CNF electrode shows exceptional areal capacitance (13 mF cm^−2^ at a current of 0.5 mA cm^−2^), good rate-retention capability (~98% retention of low-current capacitance), and long-term cycle stability for at least 5000 charge/discharge cycles. Based on the results, we believe that this electrode has potential for use as high-performance EC electrodes.

## 1. Introduction

Among the different kinds of energy storage devices, electrochemical capacitors (ECs), also termed supercapacitors, are creating new opportunities in applications in which high power, fast charge/discharge, and long-lasting operation are needed [1,2,3,4,5], because energy for ECs can be stored as the charge accumulates on the electrode surface. For high energy and power, a significant electrical conductivity and high surface area are required for electrode materials. In spite of these advantages, the relatively small energy density of ECs over those of conventional rechargeable batteries limits their commercial utilization [6,7,8,9,10]. In this regard, advances in high-energy ECs have been devoted toward the preparation of highly efficient electrodes that are a determinant of electrochemical performances [11,12]. A number of notable examples in electrode materials have been created using porous carbon nanomaterials, while maintaining their intrinsic attributes. Particularly, nanostructuring of the pores of carbon nanomaterials is significant for fast mass transports, and ion diffusion through the pores can greatly improve electrochemical reactions at the electrode/electrolyte interface [13,14,15,16]. Three-dimensional (3D) porous nanoarchitecture have been created, in which pores and active materials are interconnected, leading to fast movements for both ions and electrons [17,18,19,20]. Porous nanostructures have been commonly created through self-assembly or template-assisted strategies [21,22,23]. For self-assembly, difficulties have been encountered in forming a well-organized pore network in electrodes [24,25]. Pores are randomly distributed, sometimes not well-interconnected, and vary in size, and self-assembly is induced by randomly aggregating carbon nanomaterials [26,27,28,29]. This structure could possibly induce large ohmic resistance and incomplete wetting of electrodes by electrolytes, leading to a large voltage drop at high currents [30,31,32]. In one successful example of template-assisted methods, Choi et al. prepared 3D microporous, chemically modified graphene starting with polystyrene colloids as a sacrificial template [19]. When graphene was initially used as the electrode materials for a supercapacitor in the form of packed (or dense) film, the electrochemical performance was not significant because the electrical double layer (EDL) between the electrode and electrolyte was not well made. However, as graphene was made in a 3D structure, the pores were notably formed. This affects the diffusional (or mass transport) behavior of electrochemical systems and provides significant EDLs, which are important for capacitance, rate-dependent capability, and energy and power densities. As such, the development of a well-organized porous structure is highly desirable, yet very challenging.

One-dimensional (1D) structured carbon materials are advantageous to charge carrier mobility along the axial direction, offering the potential of being used in energy storage [33]. However, low the capacitance and energy density of 1D carbon electrodes, due to the difficulties of their pore size/size distribution controls, should be improved for practical applications [34,35,36,37,38]. Recently, studies on 1D carbon materials have been particularly driven toward the fabrication of highly porous structures with controlled pore size distributions.

Here we report nanoporous carbon nanofibers prepared by the carbonization of electrospun polyacrylonitrile (PAN), embedding monodisperse silica nanoparticles as a sacrificial template [39,40]. The chemical etching of the silica enables a nanoporous structure to form without affecting the electronic properties of PAN-based carbon nanofibers. Such a 3D porous network structure provides a facilitated transport path for electrolyte ions and electrons [41].

## 2. Materials and Methods

### 2.1. Materials

Polyacrylonitile (PAN, M_w_ = 150,000 g mol^−1^), N,N-Dimethylformamide (DMF, ≥ 99.8%, M_w_ = 73.09 g mol^−1^) and tetraethyl orthosilicate (TEOS, 98%, M_w_ = 208.33 g mol^−1^) were supplied by Sigma-Aldrich (Korea). Hydrofluoric acid (HF, M_w_ = 20.01 g mol^−1^) was supplied by J.T. Baker. Ammonium hydroxide solution (NH_4_OH, 28.0–30.0%, M_w_ = 35.05 g mol^−1^) was supplied by SAMCHUN (Korea).

### 2.2. Preparation of Porous Carbon Nanofibers

Silica nanoparticles were synthesized using the Stöber method [42]. Silica nanoparticles (500 wt% relative to polyacrylonitrile (PAN)) were mixed with 8 wt% PAN in DMF. The solution was stirred for 24 h at 50 °C and then ultrasonicated for 6 h at 50 °C to obtain a homogeneous solution. Composite nanofibers were produced via electrospinning. A 12-mL syringe with a 23-gauge metal nozzle was used for electrospinning. A high voltage supplier was used to provide a varying voltage, of 11.0, 13.0, and 14.0 kV, to the syringe needle tip and a metal drum collector (NanoNC, Korea). The electrospun composite nanofibers were collected on a metal drum collector that was covered with aluminum foil at the feed rate at 10 μL/min. The distance between the tip of the needle and the collector was 15 cm. The resulting composite nanofibers were dried in a vacuum oven for 12 h at 70 °C. Porous carbon nanofibers were formed by oxidation and subsequent carbonization of PAN/silica composite nanofibers. PAN/silica composite nanofibers were heated to 260 °C at a rate of 1 °C min^−1^ and held at that temperature for 3 h in an air atmosphere. Carbonization was performed by heating to 900 °C at a rate 2 °C min^−1^ in a N_2_ atmosphere and then the temperature was maintained for 3 h. The carbonized composite nanofibers were soaked in HF for 6 h at 25 °C to remove silica nanoparticles. The resulting porous carbon nanofibers were dried at room temperature.

### 2.3. Characterization

N_2_ adsorption/desorption isotherms were determined at 77.3 K using a surface area analyzer (ASAP 2420 V2.09K, Micromeritics, USA). Prior to measurements, the samples were preheated at 450 °C for 3 h under vacuum to remove any moisture or dust from within the pores of the samples. The Brunauer–Emmett–Teller (BET) model was used to calculate the specific surface area of the samples from the N_2_ adsorption isotherms in a relative pressure (*P/P_0_*) range of 0.02 to 0.06. Surface and cross-section images of porous carbon nanofibers were acquired by field emission scanning electron microscopy (FE-SEM, Philips XL30S FEG, Netherlands) at magnifications of ×100 to ×150 k, and at an accelerating voltage of 10 to 20 kV. Before observation, the samples were coated with a thin layer by spraying platinum (Pt) for 120 s using a Quorum Q 150T ES. Cyclic voltammetry (CV), galvanostatic charge/discharge (GCD), and electrochemical impedance spectroscopy (EIS) were performed using an SP-200 potentiostat (Bio-Logic, Knoxville, TN, USA) in 1.0 M H_2_SO_4_ aqueous electrolyte in two-electrode mode at room temperature. The EIS test was conducted over the frequency range of 10^6^ to 10^−2^ Hz at the amplitude of the sinusoidal voltage by applying a 5-mV signal. In the GCD profiles, the specific capacitance could be estimated using the following equation:

C = 4I/[(∆V/∆t)A](1)

where *I* is the current applied, ∆*V*/∆*t* is the slope of the discharge curve after the IR drop at the beginning of the discharge curve, and *A* is the total area of two electrodes.

## 3. Results and Discussion

**Figure 1:**
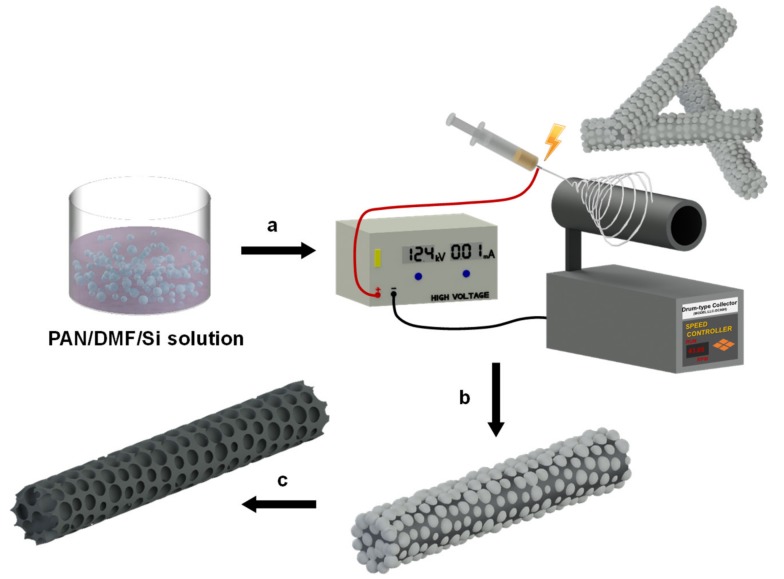
Schematic illustration of the fabrication process for porous carbon nanofibers (p-CNFs). The major steps of the fabrication process were: (**a**) Electrospinning the polyacrylonitrile (PAN)/silica solution using a metal drum collector. (**b**) Oxidation of the PAN/silica nanofibers in air at 260 °C, then carbonization at 900 °C under a slow stream of nitrogen. (**c**) Removal of silica nanoparticles by immersing in hydrofluoric acid (HF) solution for 6 h.

Porous carbon nanofibers (termed p-CNFs) were fabricated in four major steps: (1) electrospinning of polyacrylonitrile (PAN)/silica composite nanofibers; (2) oxidative stabilization at 260 °C in air; (3) carbonization at 900 °C under a nitrogen atmosphere; and (4) the removal of silica in a hydrogen fluoride aqueous solution for 6 h at room temperature (Figure 1).

**Figure 2:**
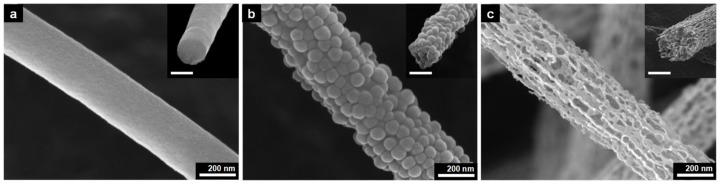
FE-SEM images of (**a**) neat CNF, (**b**) PAN/silica composite carbon nanofiber, and (**c**) p-CNF (insets: cross-sectional SEM images, scale bars = 200 nm).

Monodisperse silica nanoparticles were formed using the Stöber method [42]. The mean particle size of the silica was about 50 nm, which was determined by SEM. Neat PAN-based CNFs were also prepared using the same protocol, except for the use of silica nanoparticles. Figure 2 shows the FE-SEM images of neat PAN-based CNF, electrospun PAN/silica-basis composite carbon nanofibers, and p-CNF. Well-distributed silica nanoparticles, which were fugitive, in PAN/silica composite carbon nanofibers left pores in p-CNF after the silica etching process. As such, the p-CNF had pores evenly distributed in the fibers. This was due to the good dispersion of the silica nanoparticles in the PAN solution. Fiber diameters ranging from 250 to 300 nm were preserved after the carbonization and etching processes.

**Figure 3:**
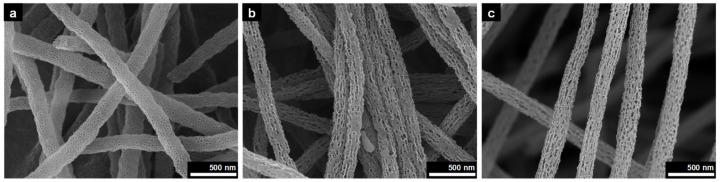
FE-SEM images of p-CNFs prepared by 8 wt% PAN dissolved in DMF solution with (**a**) 350 wt%, (**b**) 500 wt%, and (**c**) 700 wt% silica nanoparticle contents (insets: cross-sectional SEM images, scale bars = 200 nm).

Porosity of the p-CNF was adjusted by changing the wt% of silica nanoparticles to PAN ranging from 350 to 700 wt% because it depended on the amounts of silica nanoparticles that adhered to the CNF surface. Figure 3 shows FE-SEM images of p-CNFs prepared using different silica nanoparticles contents. Not surprisingly, the porosity of the p-CNFs increased by increasing the content of silica nanoparticles from 350 to 500 wt%. No significant change in porosity of p-CNF was observed when 750 wt% of silica nanoparticles were added to the PAN solution when compared to 500 wt% silica content. Accordingly, we limited the discussion here to p-CNF prepared by using PAN solution with 500 wt% silica nanoparticles.

Pore characteristics of CNF and p-CNF were investigated using N_2_ sorption measurements. The specific surface area, micropore area, pore volume and pore size are summarized in Table 1. The uniformly dispersed silica nanoparticles in the CNF offered numerous channels to interconnect with the micropores, leading to an increase in the surface area. As a result, the p-CNF had a specific surface area of 391 m^2^ g^−1^, which isapproximately 1.8 times higher than that of CNF (214 m^2^ g^−1^). The pore volume of p-CNF determined by Barrett–Joyner–Halenda (BJH) pore size distribution was also larger than CNF. These observations are presumably due to pores that were formed after removal of the silica nanoparticles.

Judging from these data, it can be concluded that the silica nanoparticles were successfully introduced into the CNF without any structural collapse. Furthermore, the interconnected pore structure of p-CNF with a high surface area was expected to increase wettability of electrolyte, and thus enable them to be used as highly efficient electrode for ECs.

**Figure 4:**
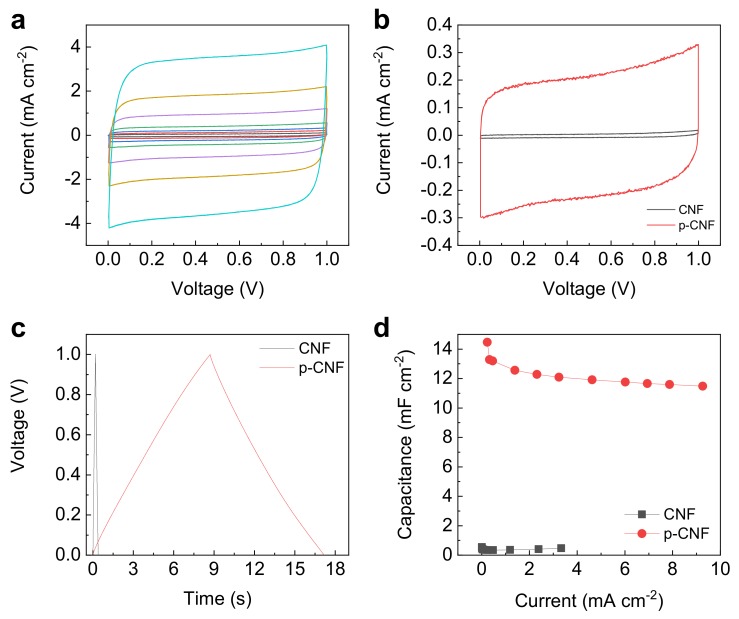
(**a**) Cyclic voltammetry (CV) curves of p-CNF electrode at scan rates of 10 to 1000 mV s^−1^. (**b**) CV curves (at a constant scan rate of 50 mV s^−1^), (**c**) galvanostatic charge/discharge (GCD) profiles (at a constant current of 0.5 mA cm^−2^), and (**d**) rate-retention capability of CNF and p-CNF electrodes.

The electrochemical characterizations of p-CNF and CNF electrodes were carried out by two-electrode cyclic voltammetry (CV) and galvanostatic charge/discharge (GCD) tests over the voltage window of 1 V in 1.0 M H_2_SO_4_ aqueous electrolyte at room temperature. Figure 4a shows the CV curves of p-CNF and CNF electrodes, both of which retain nearly box-like curves even up to a high scan rate of 1000 mV s^−1^. This is indicative of excellent charge propagation across electrodes. Additionally, the current induced by charge accumulation for p-CNF is significantly larger than that of CNF at a constant scan rate of 50 mV s^−1^ (Figure 4b). The corresponding areal capacitance is 38 mF cm^−2^, 37-fold greater than 1.0 mF cm^−2^ of CNF. This observation was supported by performing GCD measurements over the currents of 0.04 to 9.3 mA cm^−2^. A longer triangular GCD profile of p-CNT electrode than that of a CNF electrode demonstrated that the former electrodes accumulated the larger amount of EDLs on the surface (Figure 4c). The areal capacitance of p-CNF is 13 mF cm^−2^, 38-times larger than 0.34 mF cm^−2^ of CNF, which was comparable to values of other state-of-the-art electrical double layer capacitor (EDLC) type electrodes reported [43,44,45]. Fast charge–discharge behavior within seconds was also observed, leading to high power capability. Rate-retention performance was obtained by calculating the discharge curve of the GCD (Figure 4d). The areal capacitance of 14 mF cm^−2^ for p-CNF at low current of 0.23 mA cm^−2^ was retained by 79% even at a large areal-based current of 9.3 mA cm^−2^, proving exceptional rate-retention capability.

**Figure 5:**
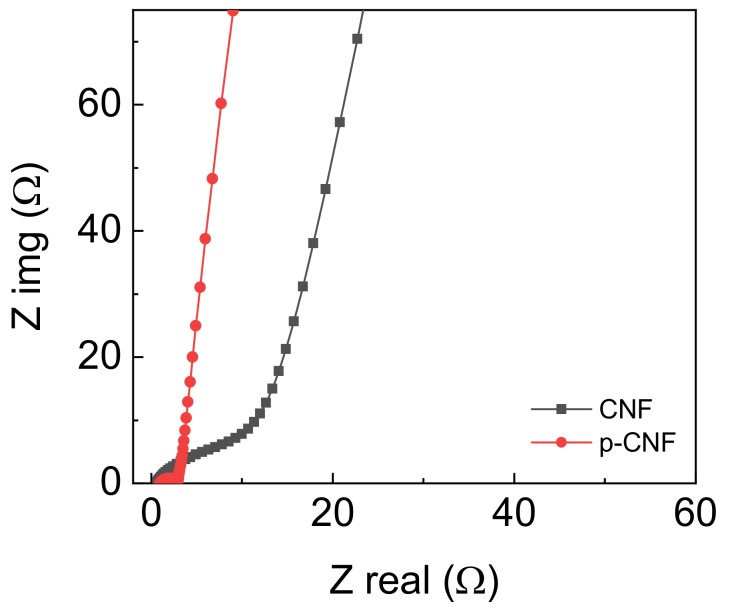
Nyquist plots of CNF and p-CNF electrodes.

The additional details of why p-CNF exhibited better performance than CNF were identified through electrochemical impedance spectroscopy measurements over a frequency range of 10^6^ to 10^−2^ Hz (Figure 5). The intercept of the real part of the Nyquist plot was related to the equivalence series resistance (ESR) that was attributed to the combination of electrode and electrolyte resistances and contact resistance between the current collector and electrode. The ESR for p-CNF was quite small, 3.0 Ω, close to 1.1 Ω of CNF, which indicated that pore formation via a silica template did not aggravate the electrical property of p-CNF. The intermediate frequency region of the Nyquist plots, i.e., 45° Warburg region, exhibited the slope deviated from the vertical line of ideal resistor-capacitor (RC) circuit, attributed to the limited ion diffusion of the electrolyte [46]. The p-CNF electrodes displayed much shorter Warburg length than CNF electrodes, and demonstrated faster ion diffusion across electrode/electrolyte interface due to the porous pores in p-CNF [47,48]. The nonporous CNF electrode resulted in relatively longer Warburg length, reflecting slow ion kinetics. The more vertical slope of the p-CNF electrode than that of the CNF electrode in the low-frequency region indicated a purely capacitive behavior. With the results we observed, we can conclude that better electrochemical performances of the p-CNF electrode than the neat CNF electrode were due to the electrode/electrolyte interface largely formed throughout the porous nanofiber electrodes.

**Figure 6:**
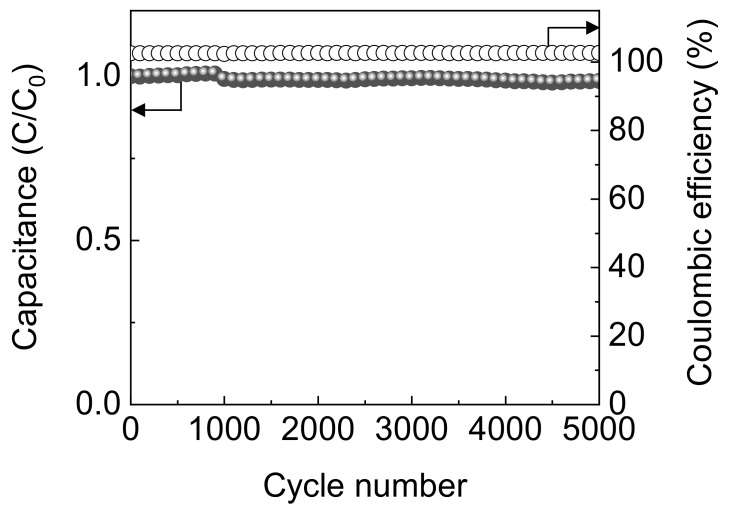
Long-term cycle stability and coulombic efficiency of p-CNF over 5000 GCD cycles at 0.19 mA cm^−^^2^. *C*_0_ is the initial capacitance and *C* is the capacitance at the indicated number of cycles.

Long-term cycle stability of the p-CNF electrode was confirmed over repeated 5000 GCD cycles at a constant current of 0.19 mA cm^−2^ (Figure 6). The electrode retained ~98% of initial areal capacitance and nearly 100% of coulombic efficiency for at least 5000 GCD cycles, demonstrating good cycle stability.

## 4. Conclusions

In conclusion, the present work demonstrated that the concept of porous carbon design holds great potential as an electrochemical energy storage. Starting from electrospun PAN/silica composites, p-CNFs were formed via oxidation/carbonization and subsequent silica etching. The porosity of p-CNFs was modulated by a simple change of wt% of PAN to silica nanoparticles. The optimum weight% was found to be 500 wt%. Cyclic voltammetry and galvanostatic charge/discharge results demonstrated that p-CNF electrodes revealed better electrochemical performances with respect to areal capacitance and rate performance than neat CNF electrodes. This relatively high-performance of p-CNF was due to enhanced wettability of electrode by electrolyte through well-connected pores, synergistically improving the effective surface area of the electrode/electrolyte interface. Additionally, they exhibited exceptional long cycle lives and coulombic efficiency for at least 5000 charge/discharge cycles.

## Figures and Tables

**Table 1:** Pore characteristics of CNF and p-CNF.

Samples	Specific Surface Area ^1^ (m^2^/g)	Micropore Area ^2^ (m^2^/g)	Pore Volume ^3^ (cm^2^/g)	Pore Size ^1^ (nm)
CNF	214.19	198.74	0.118	2.218
p-CNF	391.43	260.22	0.987	10.088

^1^ These values are estimated by using the Brunauer–Emmett–Teller (BET) model. ^2^ Estimated by t-plot method. ^3^ These values are estimated by using the Barrett–Joyner–Halenda (BJH) model.
